# Supplementary material for: Investigations on the Influence of the Axial Ligand in [Salophene]iron(III) Complexes on Biological Activity and Redox Behavior
Source: Int J Mol Sci. 2023 Jan 21;24(3):2173. doi: 10.3390/ijms24032173 (PMC9916684; doi:10.3390/ijms24032173)

Investigation of axial ligand exchange of iron(III) salophene complexes

Hubert Descher,[1] Sophie Luise Strich,[2,3] Martin Hermann,[4] Peter Enoh,[1] Brigitte Kircher, [*2,3, †]and Ronald Gust[*1,†]

[1] Department of Pharmaceutical Chemistry, Institute of Pharmacy, CMBI – Center for Molecular Biosciences Innsbruck, CCB – Center for Chemistry and Biomedicine, University of Innsbruck, Innrain 80-82, 6020 Innsbruck, Austria.

[2] Tyrolean Cancer Research Institute, Innrain 66, 6020 Innsbruck, Austria.

[3] Immunobiology and Stem Cell Laboratory, Department of Internal Medicine V (Hematology and Oncology), Innsbruck Medical University, Anichstraße 35, 6020 Innsbruck, Austria.

[4] Department of Anesthesiology and Critical Care Medicine, Innsbruck Medical University, Anichstraße 35, 6020 Innsbruck, Austria.

* Correspondence: brigitte.kircher@i-med.ac.at; ronald.gust@uibk.ac.at

Tel: +43-512-504-82142 (B.K.); +43-512-507-58200 (R.G.)

† These authors contributed equally.

Table of Content: Supporting information

1 Characterization 2
- 1.1 HPLC method 2
- 1.2 HPLC chromatograms 3
- 1.3 HR-MS spectra 7
- 1.4 FT-IR spectra. 11

2 Biological data 16
- 2.1 Proliferation 16
- 2.2 Metabolic activity 16
- 2.3 Cell-death induction 17
- 2.4 ROS 18
- 2.5 Mitochondrial membrane potential 19

3 Cyclic voltammetry 20
- 3.1 Oxygen source for the formation of compound **7** 20
- 3.2 Voltammograms of the complexes **1–7** 21

1 Characterization

1.1 HPLC method

HPLC method was conducted similarly to previously published work. All complexes were solved via ultrasound bath in methanol (complexes **1-6**) or acetonitrile (complex **7**) to obtain a 0.5 mM solution. Running solvent was 35% methanol / 65% phosphate buffer solution (25 mM sodium dihydrogen phosphate; pH = 3 adjusted with phosphoric acid). Flow rate was 1 ml / min. Running time per complex was 20 minutes at 25 °C.

All of the complexes showed very similar retention times (see **Table S1**). Explanation for this behavior are probably exchange reaction of the axial ligand with the phosphate buffer.

Table S1: Overview of the HPLC retention times of compounds 1–7.

Compound	Retention time (Complex)
**1**	4.34 minutes
**2**	4.31 minutes
**3**	4.32 minutes
**4**	4.33 minutes
**5**	4.30 minutes
**6**	4.30 minutes
**7**	4.32 minutes

1.2 HPLC chromatograms

Figure S1: HPLC chromatogram of **1**.

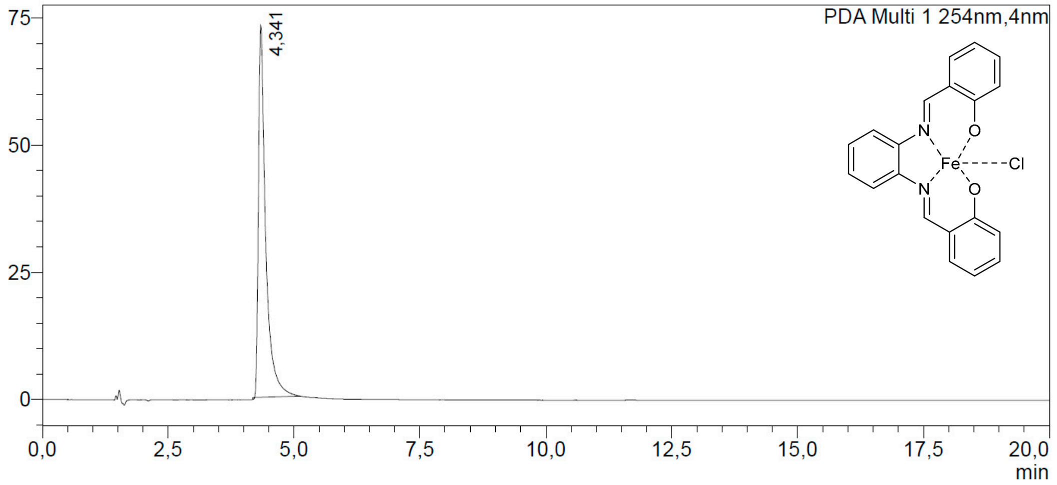

<Peak Table>

PDA Ch1 254nm

Peak#	Ret. Time	Area	Height	Conc.	Unit	Mark	Name
1	4,341	733017	72911	100,000		M	
Total		733017	72911				

Figure S2: HPLC chromatogram of **2**.

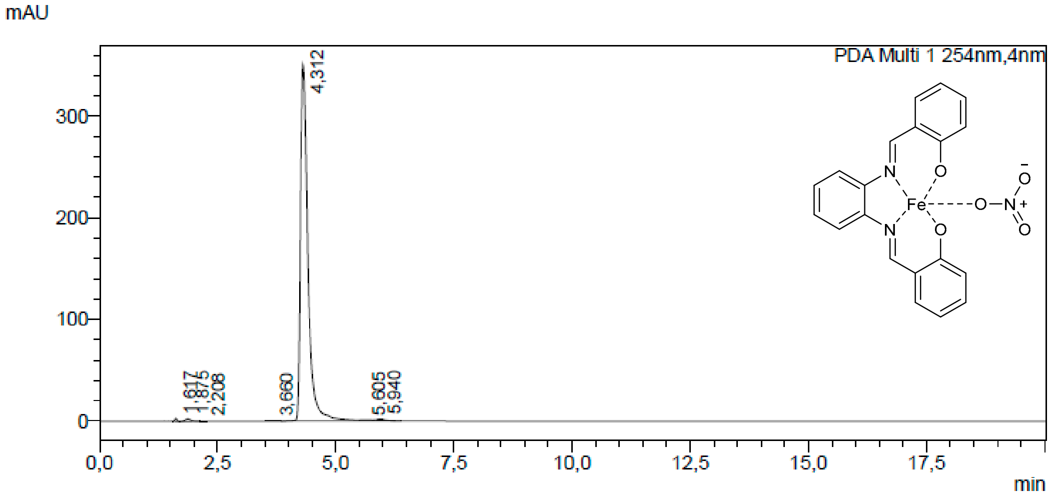

<Peak Table>

PDA Ch1 254nm

Peak#	Ret. Time	Area	Height	Conc.	Unit	Mark	Name
1	1,617	8268	2869	0,210			
2	1,875	23678	2595	0,601			
3	2,208	1506	171	0,038		V	
4	3,660	1755	184	0,045			
5	4,312	3888477	350153	98,694		SV	
6	5,605	1770	209	0,045		T	
7	5,940	14478	1561	0,367		T	
Total		3939932	357742				

Figure S3: HPLC chromatogram of **3**.

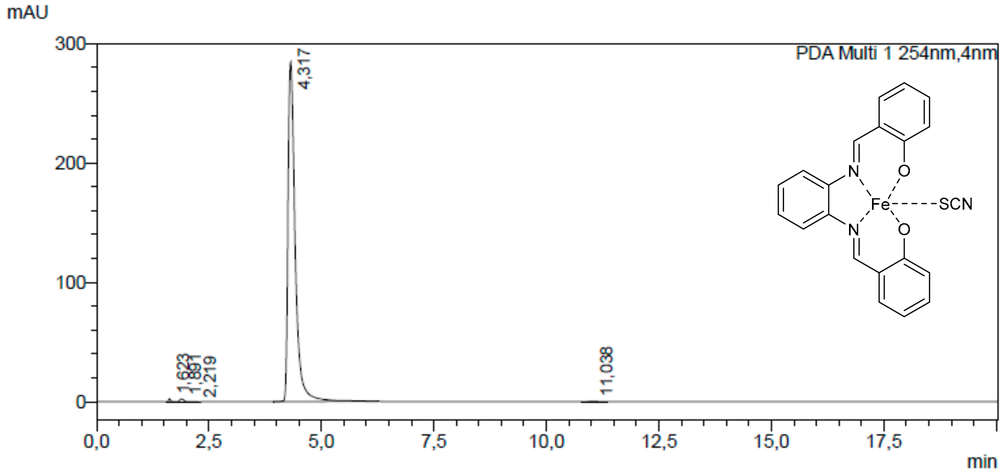

<Peak Table>

PDA Ch1 254nm

Peak#	Ret. Time	Area	Height	Conc.	Unit	Mark	Name
1	1,623	7977	2634	0,249			
2	1,891	16469	2439	0,514		V	
3	2,219	1159	139	0,036		V	
4	4,317	3170584	284229	98,989		S	
5	11,038	6762	470	0,211			
Total		3202950	289911				

Figure S4: HPLC chromatogram of **4**.

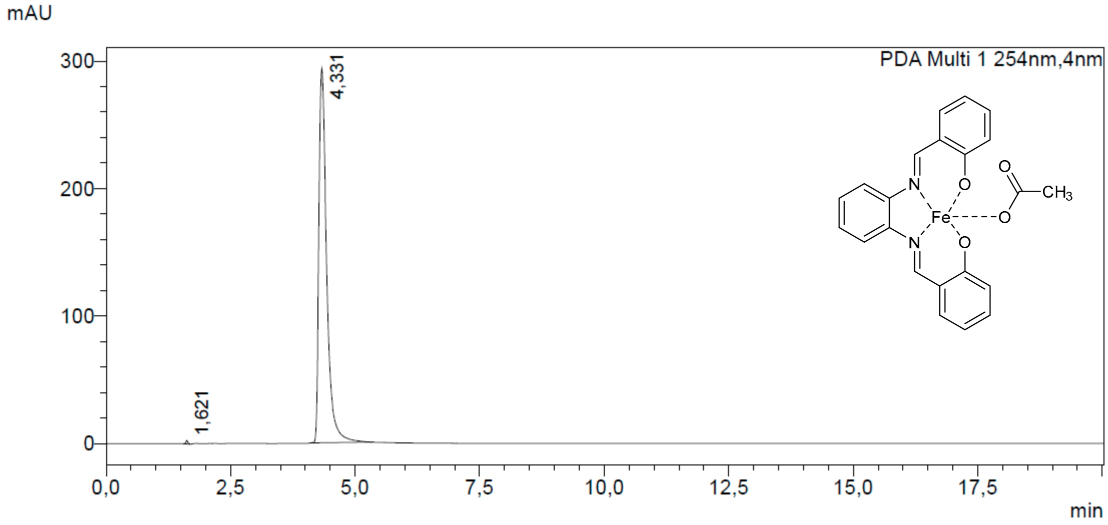

<Peak Table>

PDA Ch1 254nm

Peak#	Ret. Time	Area	Height	Conc.	Unit	Mark	Name
1	1,621	6948	2690	0,217			
2	4,331	3198587	293355	99,783		M	
Total		3205535	296046				

Figure S5: HPLC chromatogram of **5**.

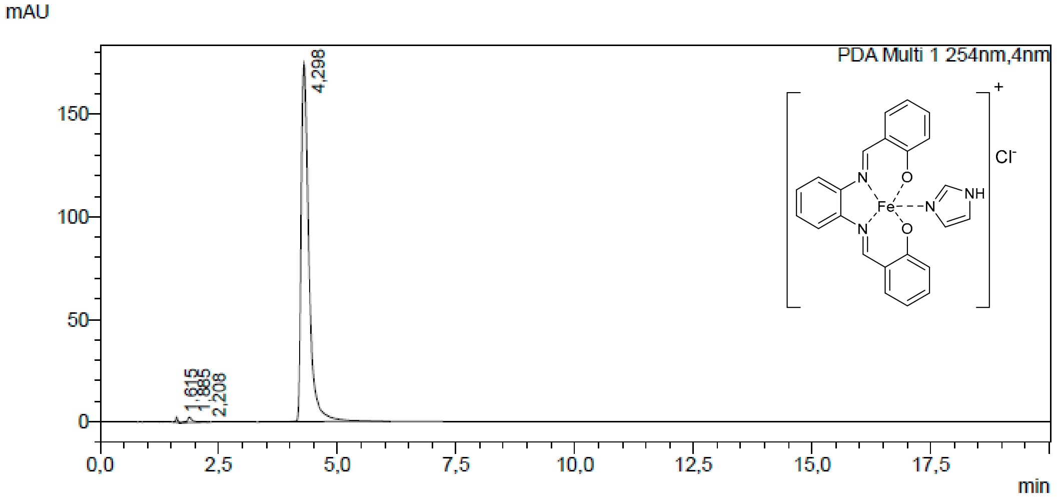

<Peak Table>

PDA Ch1 254nm

Peak#	Ret. Time	Area	Height	Conc.	Unit	Mark	Name
1	1,615	7438	2675	0,370			
2	1,885	20649	2619	1,029			
3	2,208	1547	169	0,077		V	
4	4,298	1977930	174144	98,524		S	
Total		2007563	179606				

Figure S6: HPLC chromatogram of **6**.

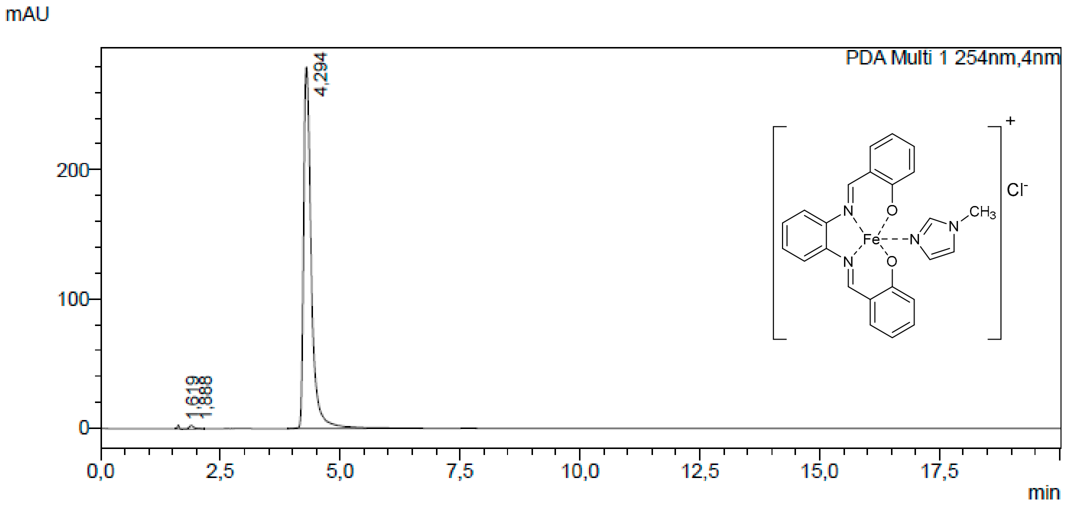

<Peak Table>

PDA Ch1 254nm

Peak#	Ret. Time	Area	Height	Conc.	Unit	Mark	Name
1	1,619	7795	2768	0,249			
2	1,888	22041	2768	0,703			
3	4,294	3104365	278943	99,048		S	
Total		3134201	284480				

Figure S7: HPLC chromatogram of **7**.

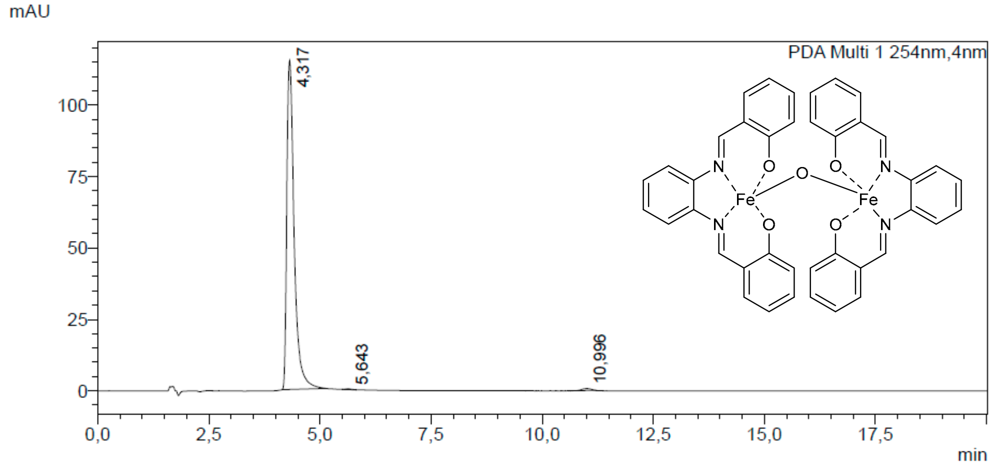

<Peak Table>

PDA Ch1 254nm

Peak#	Ret. Time	Area	Height	Conc.	Unit	Mark	Name
1	4,317	1323944	115103	99,122		M	
2	5,643	2381	244	0,178		M	
3	10,996	9353	700	0,700		M	
Total		1335678	116047				

1.3 HR-MS spectra

Scheme S1: Fragmentation during electrospray ionisation.

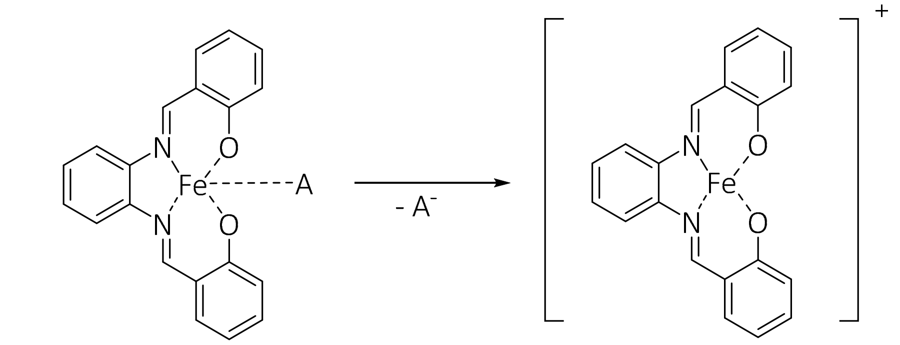

A[salophene]iron(III)

[salophene]iron(III)$^{+}$ = 370.0399 (m/z)

Figure S8: HR-MS spectrum of **1**.

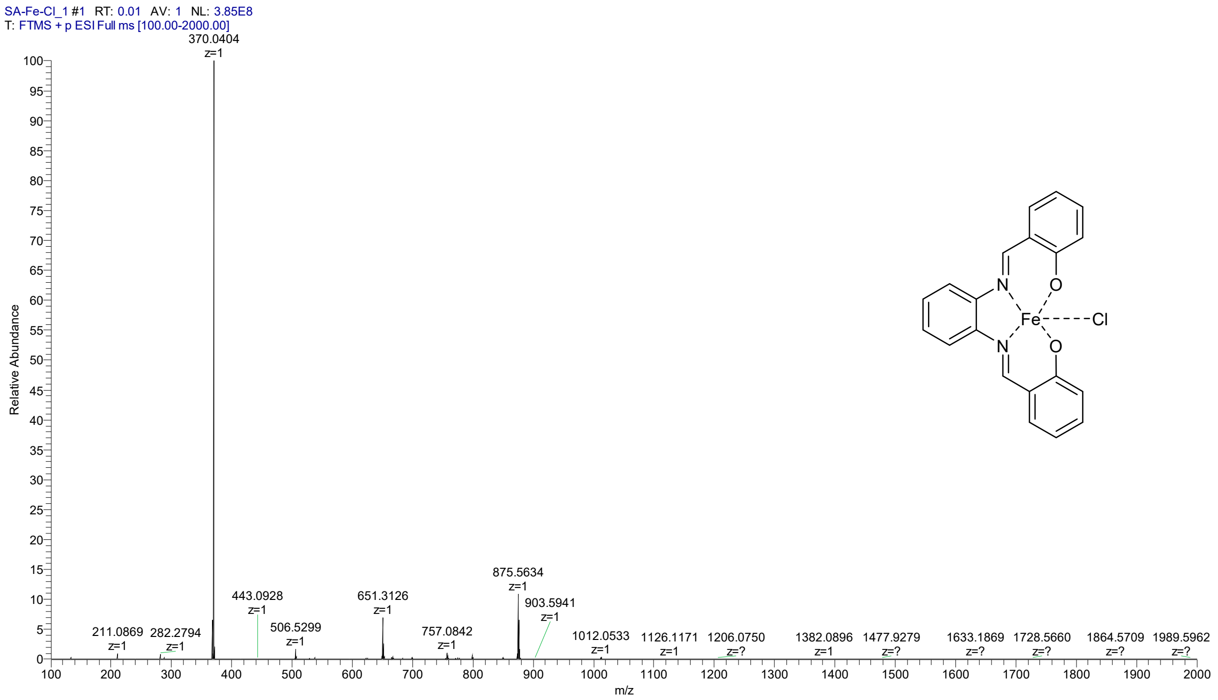

Figure S9: HR-MS spectrum of 2.

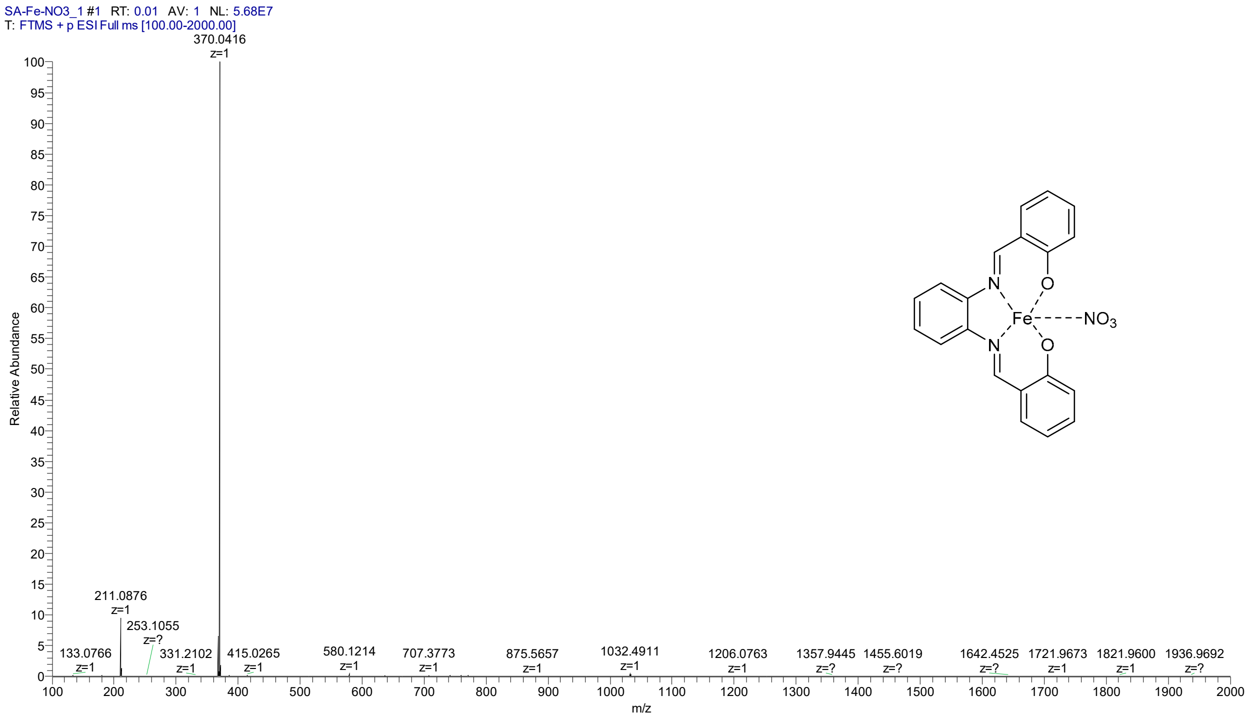

Figure S10: HR-MS spectrum of 3.

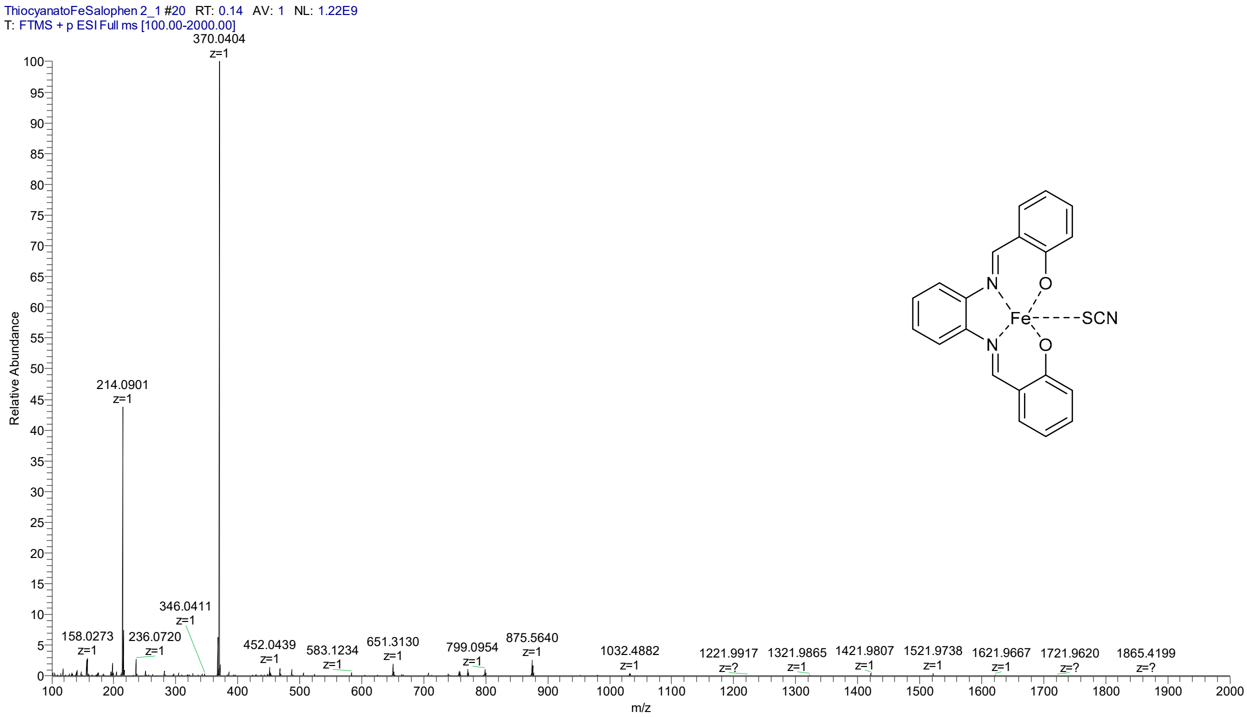

Figure S11: HR-MS spectrum of **4**.

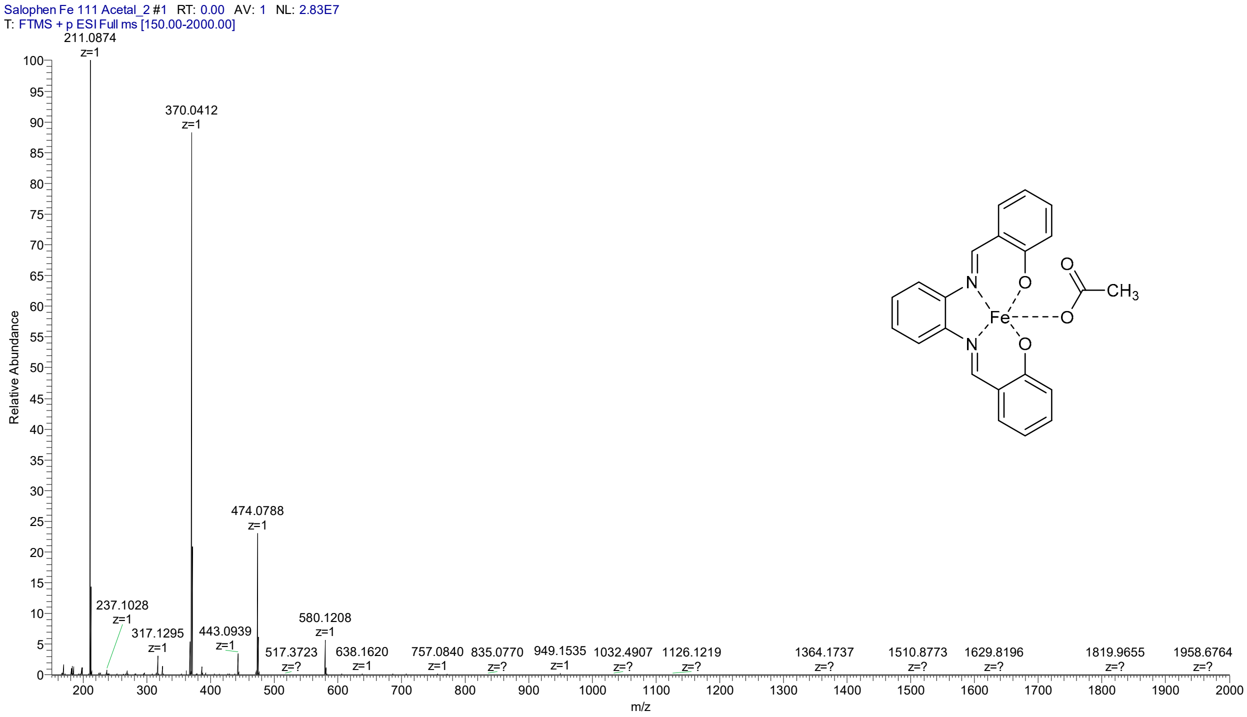

Figure S12: HR-MS spectrum of **5**.

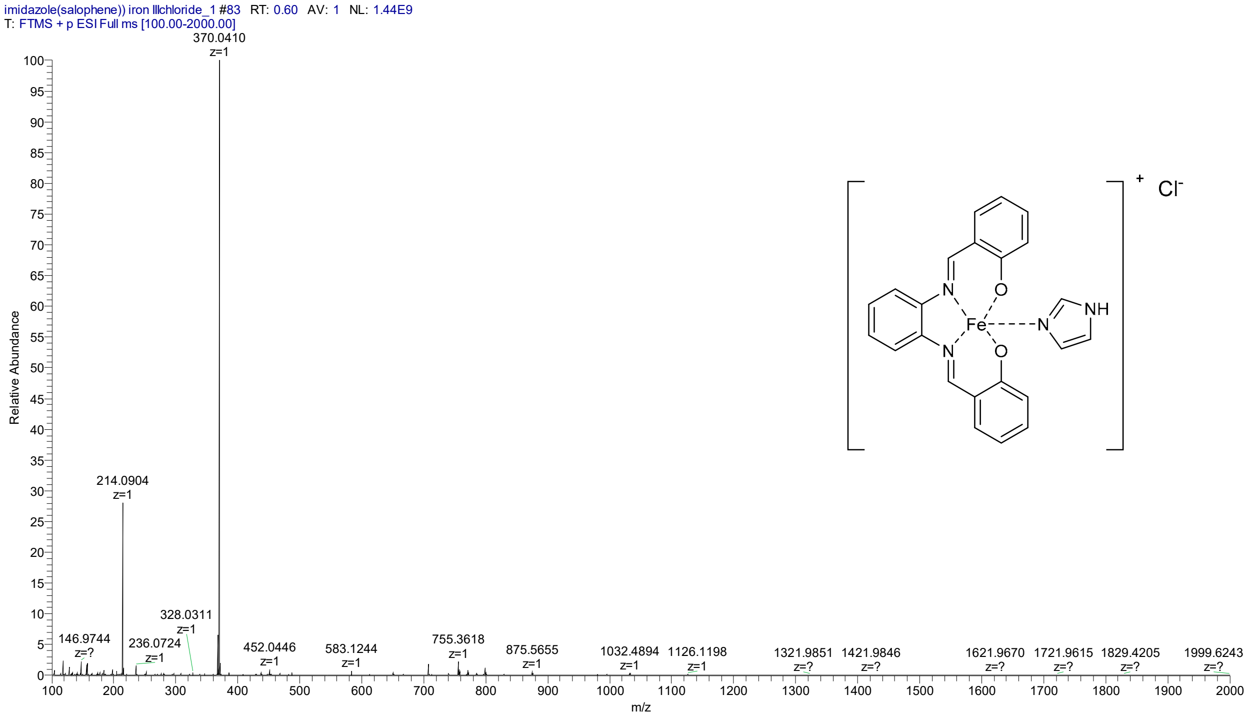

Figure S13: HR-MS spectrum of 6.

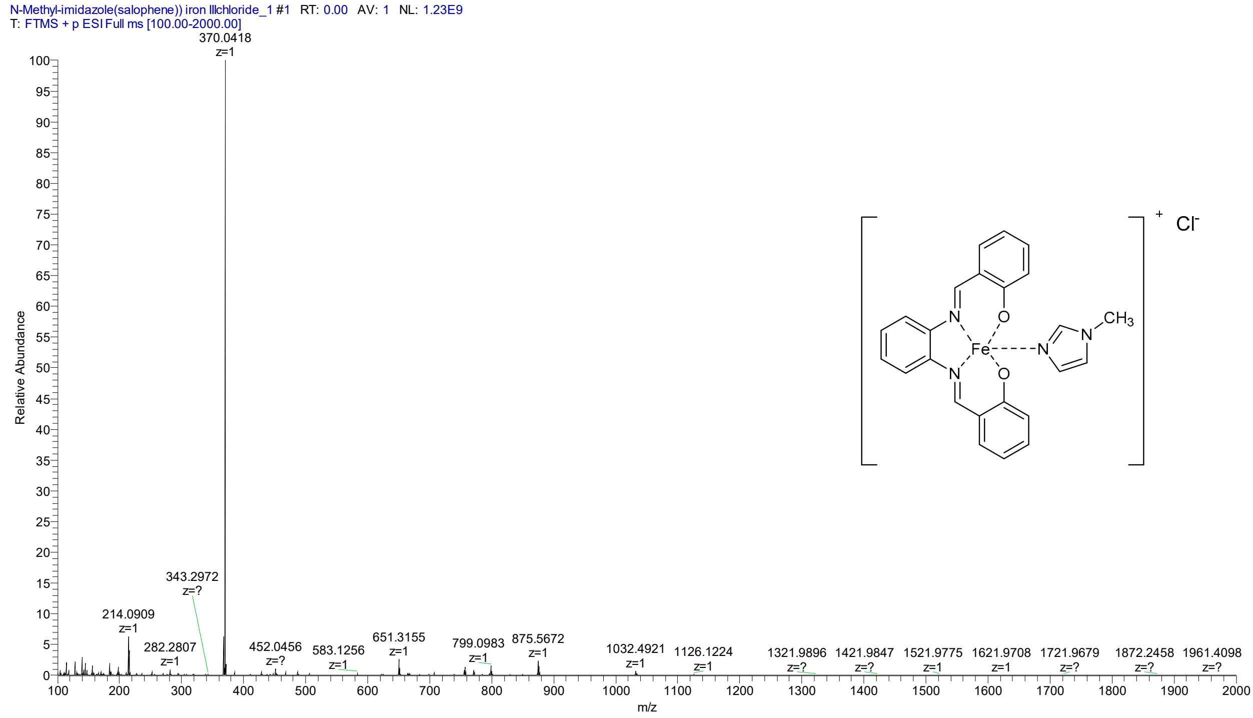

Figure S14: HR-MS spectrum of 7.

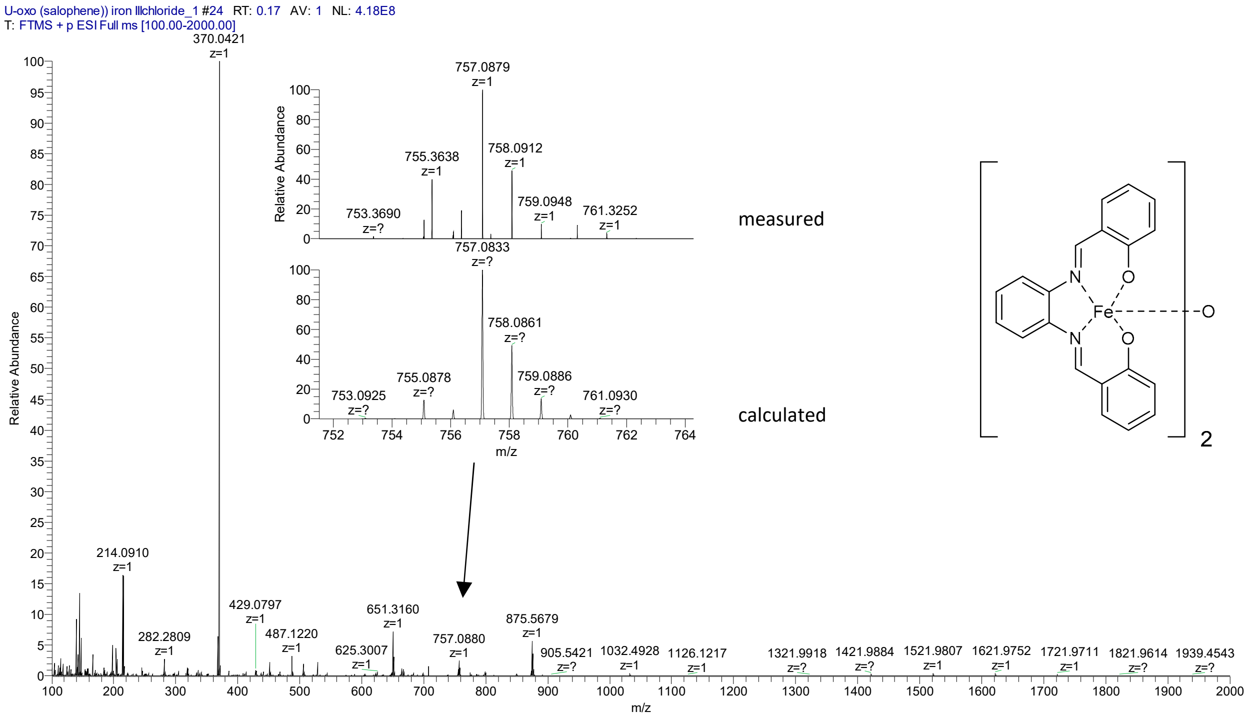

1.4 FT-IR spectra.

Figure S15: FT-IR spectrum of *N,N'*-disalicylidene-1,2-phenylenediamine.

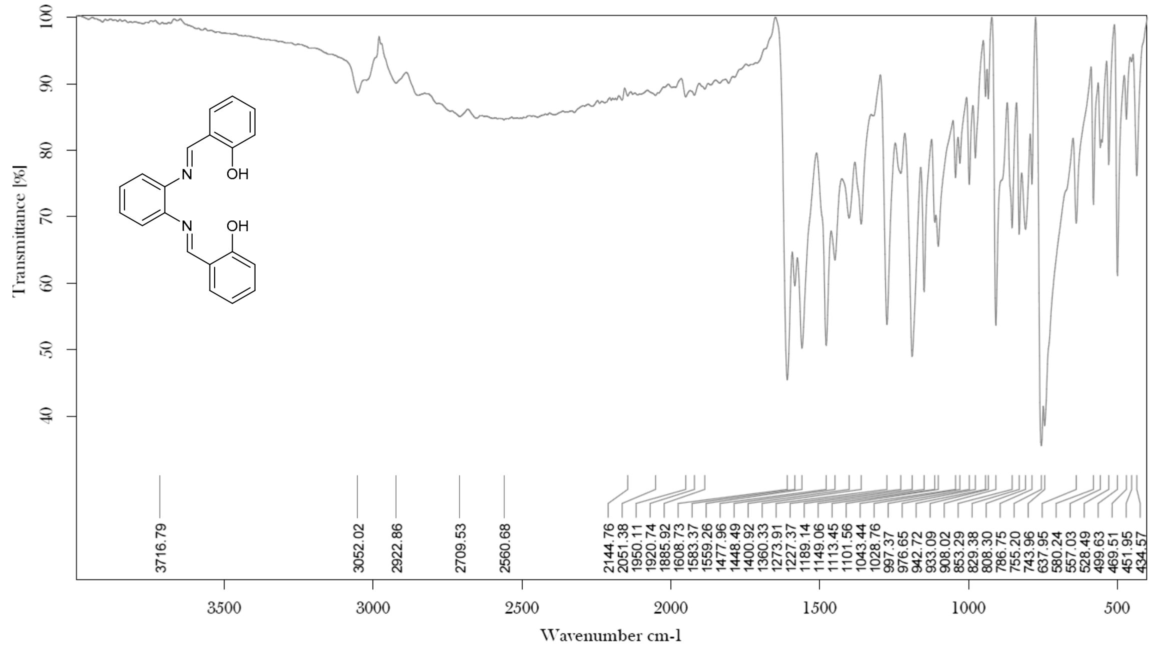

Figure S16: FT-IR spectrum of iron(III)thiocyanate.

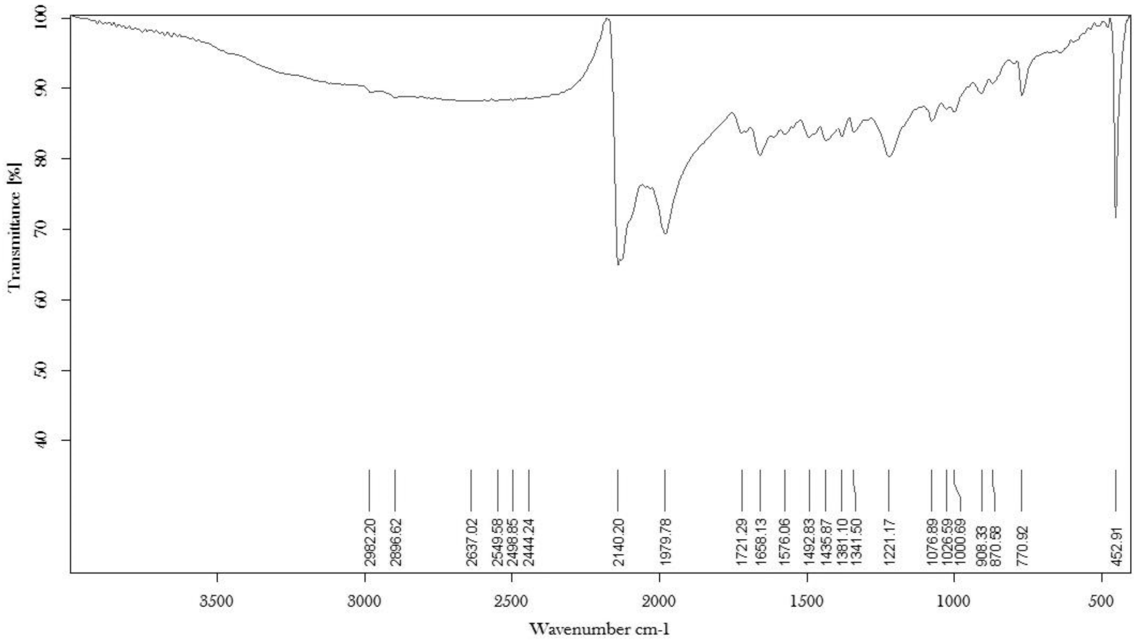

Figure S17: FT-IR spectrum of 1.

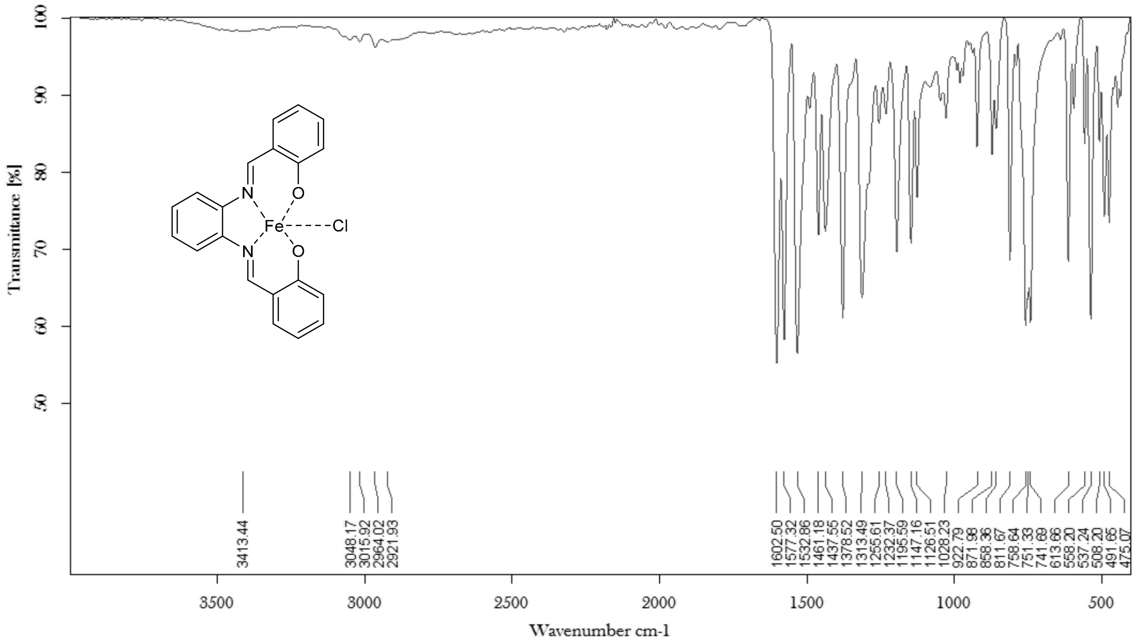

Figure S18: FT-IR spectrum of 2.

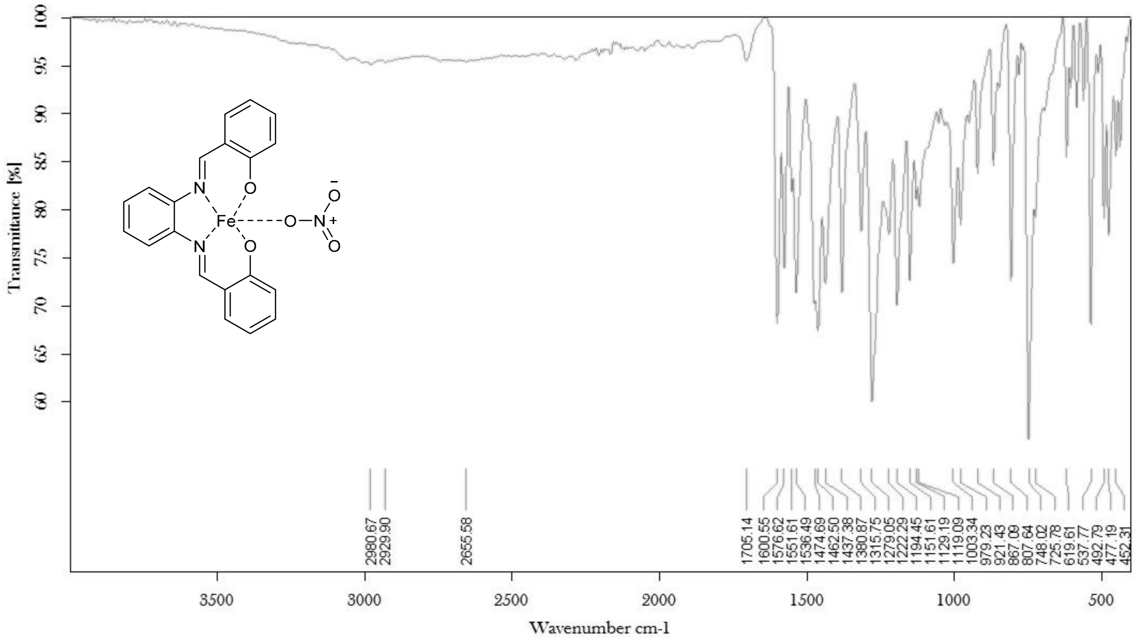

Figure S19: FT-IR spectrum of **3**.

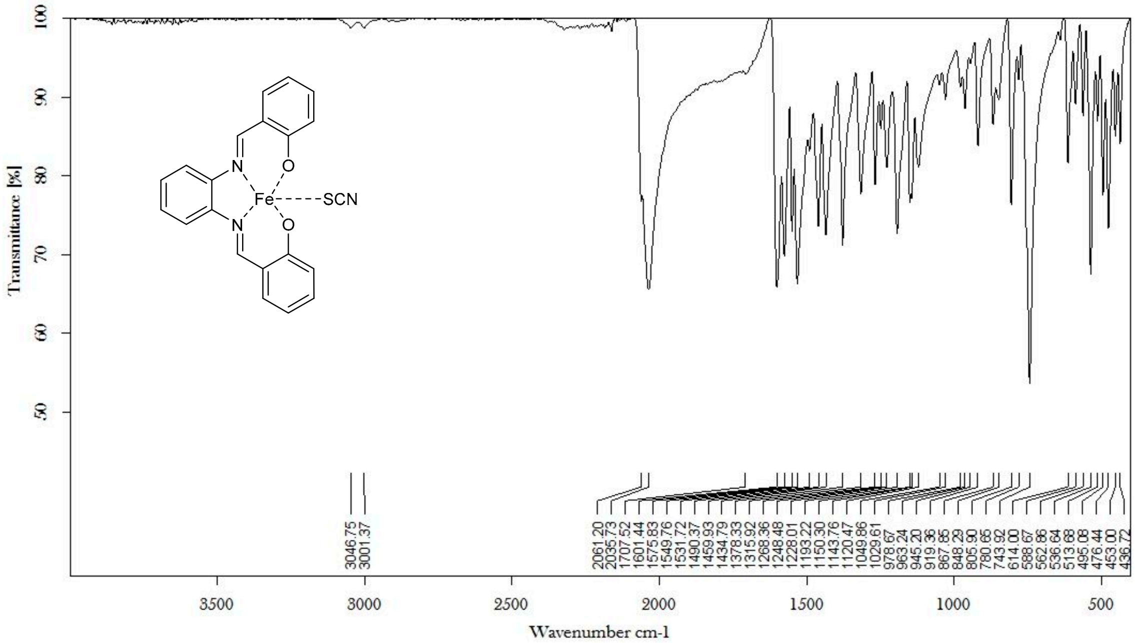

Figure S20: FT-IR spectrum of **4**.

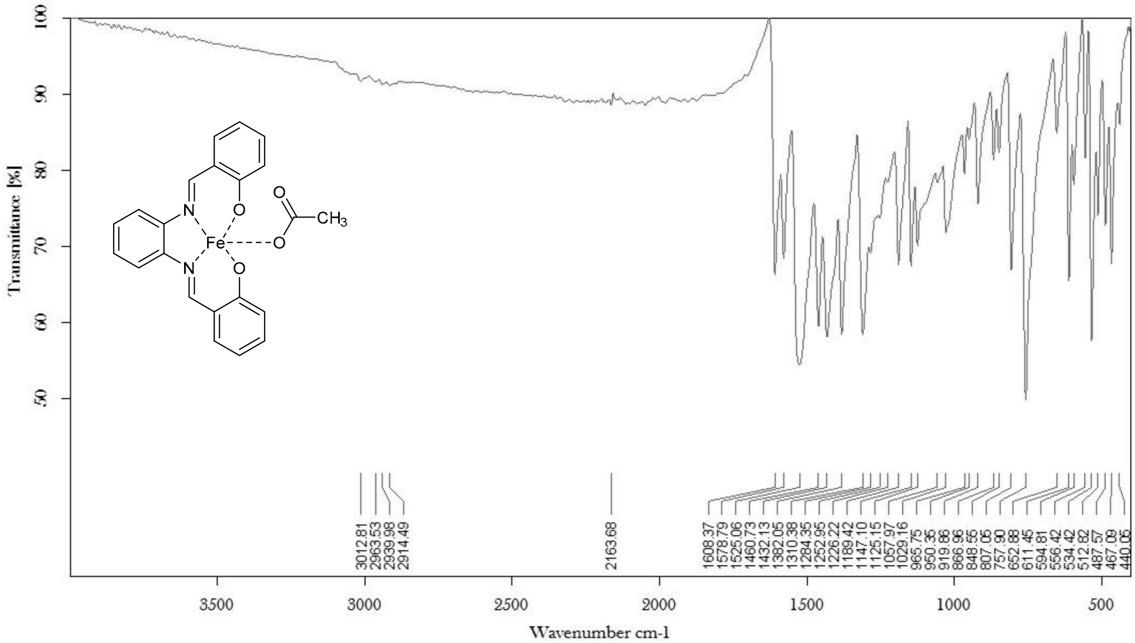

Figure S21: FT-IR spectrum of **5**.

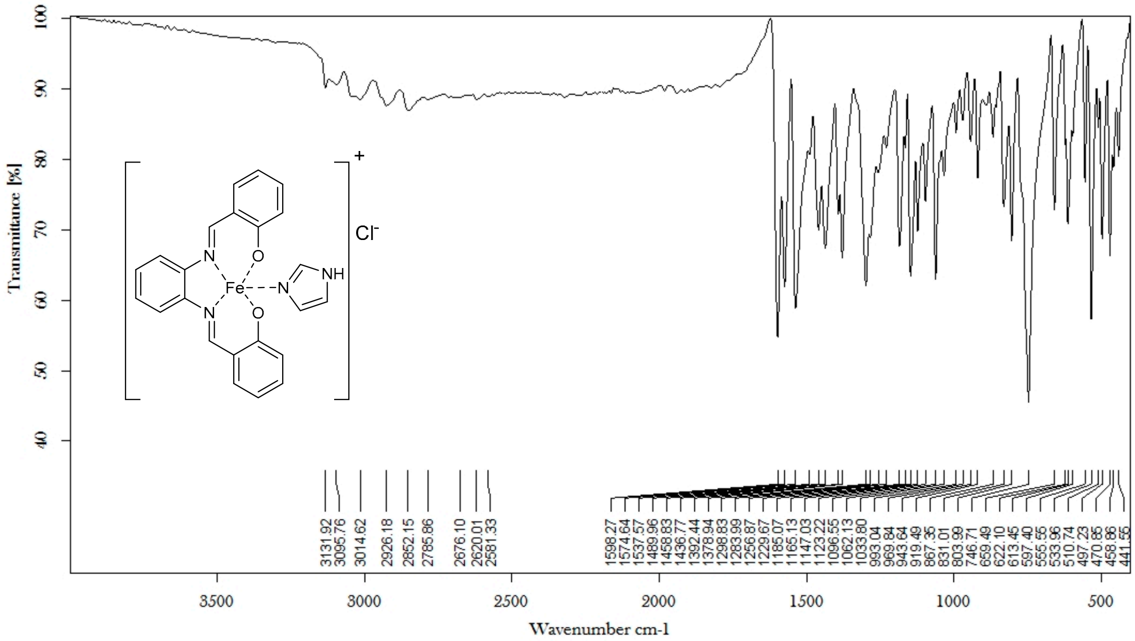

Figure S22: FT-IR spectrum of **6**.

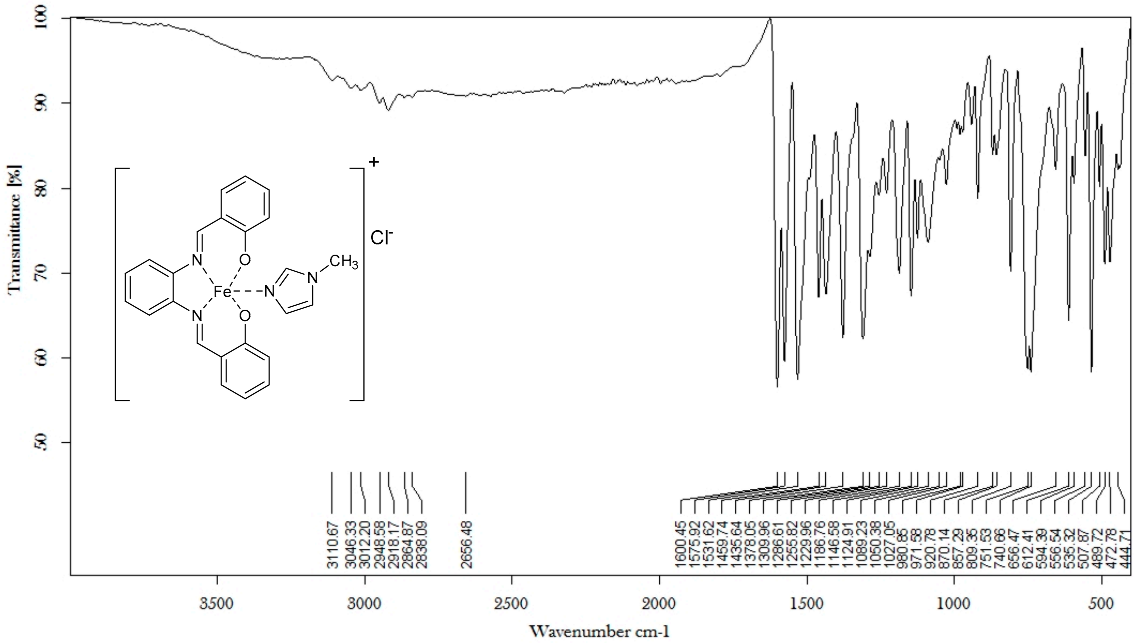

Figure S23: FT-IR spectrum of 7.

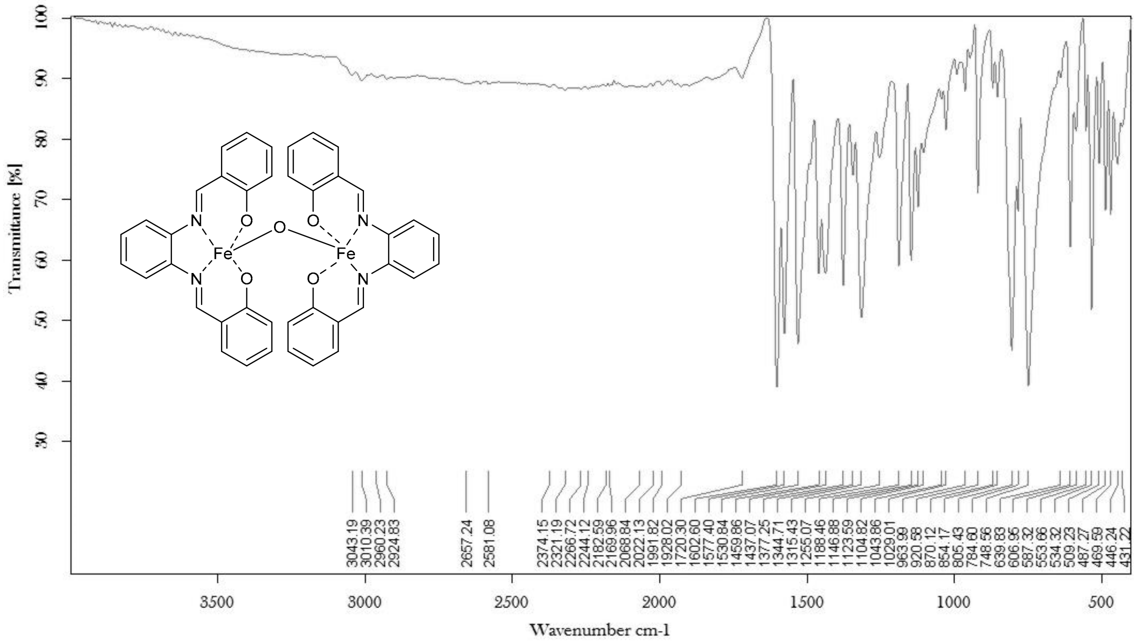

2 Biological data

2.1 Proliferation

Figure S24: Proliferation of MDA-MB231 cells treated with the complexes **1-7** at concentrations of 0.05 µM (light yellow), 0.1 µM (medium yellow), 0.5 µM (light ochre yellow) and 1 µM (dark ochre yellow). Data are expressed as mean + SE of 5 experiments.

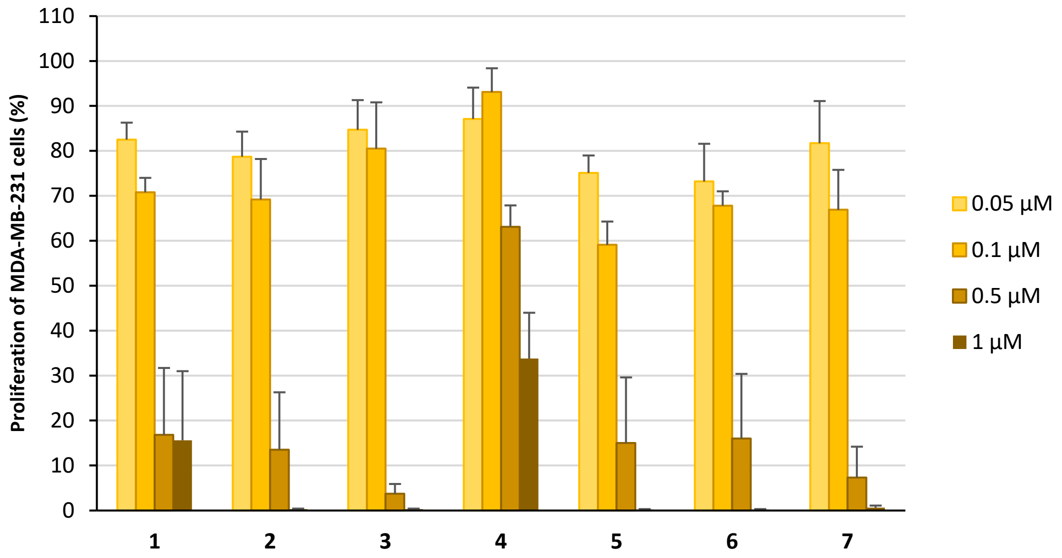

2.2 Metabolic activity

Figure S25: Metabolic activity of MDA-MB231 cells treated with the complexes **1-7** at concentrations of 0.05 µM (light yellow), 0.1 µM (medium yellow), 0.5 µM (light ochre yellow), 1 µM (dark ochre yellow) and 5 µM (dark brown). Data are expressed as mean + SE of 5 experiments.

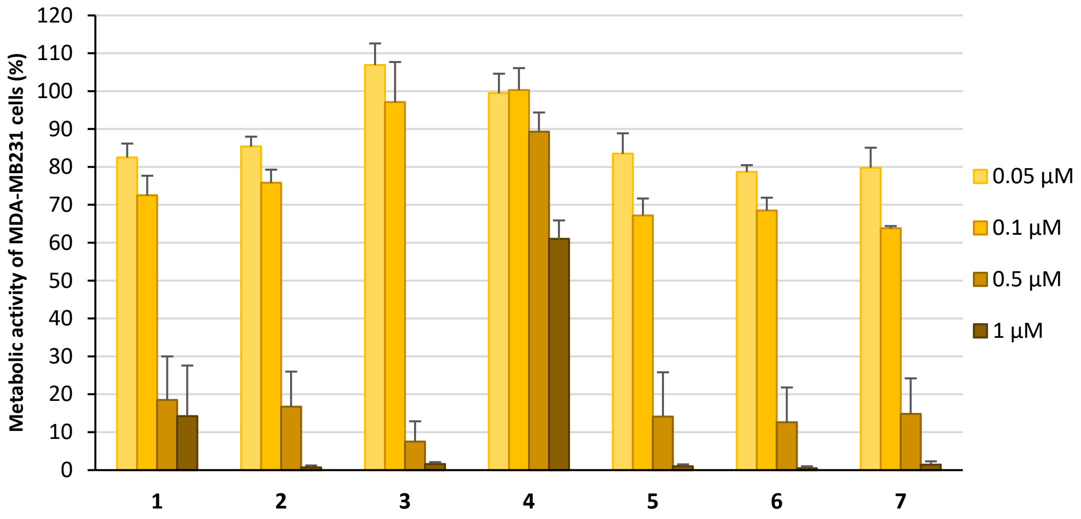

2.3 Cell-death induction

Table S2: Induction of caspases 3/7 activity in A2780cis cells after incubation with **1-7** (1 µM) for 24 h. Caspases 3/7 activity in the absence of the compounds was set at 1. Data are expressed as mean ± SE of 5 experiments.

Compound	Mean caspases 3/7 activity ± SE [x-fold]
**1**	2.5 ± 0.7
**2**	3.1 ± 0.7
**3**	3.3 ± 0.7
**4**	2.0 ± 0.3
**5**	3.1 ± 0.6
**6**	3.5 ± 0.6
**7**	2.6 ± 0.6

Figure S26: Differentiation of the MDA-MB-231 cell population (%) into apoptotic (dark blue), dead (grey) and cells alive (blue), respectively, after incubation with **1**, **2** and **5** (1 µM) for 24 h. Data are expressed as mean of 2 experiments.

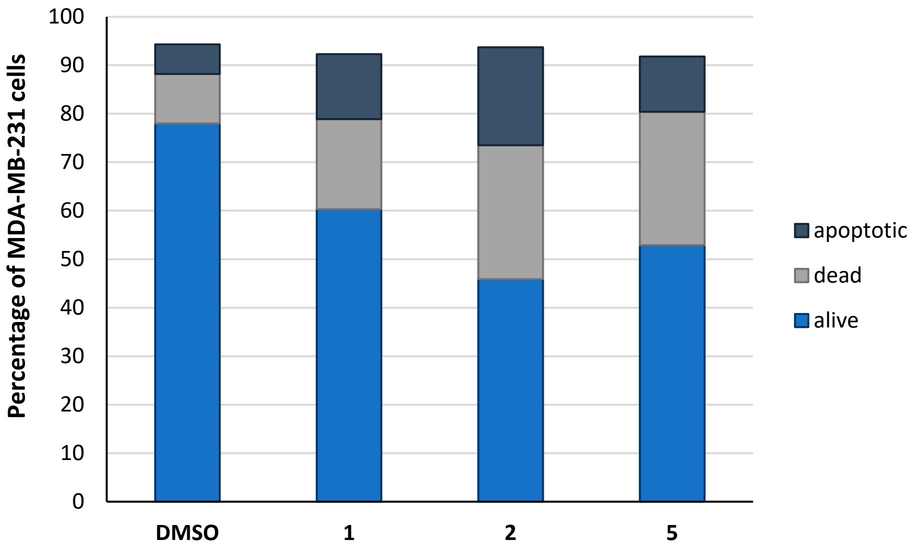

Figure S27: Differentiation of the MDA-MB-231 cell population (%) after Ferrostatin-1 treatment (1 µM) into apoptotic (dark blue), dead (grey) and cells alive (blue), respectively, after incubation with **1**, **2** and **5** (1 µM) for 24 h. Data are expressed as mean of 2 experiments.

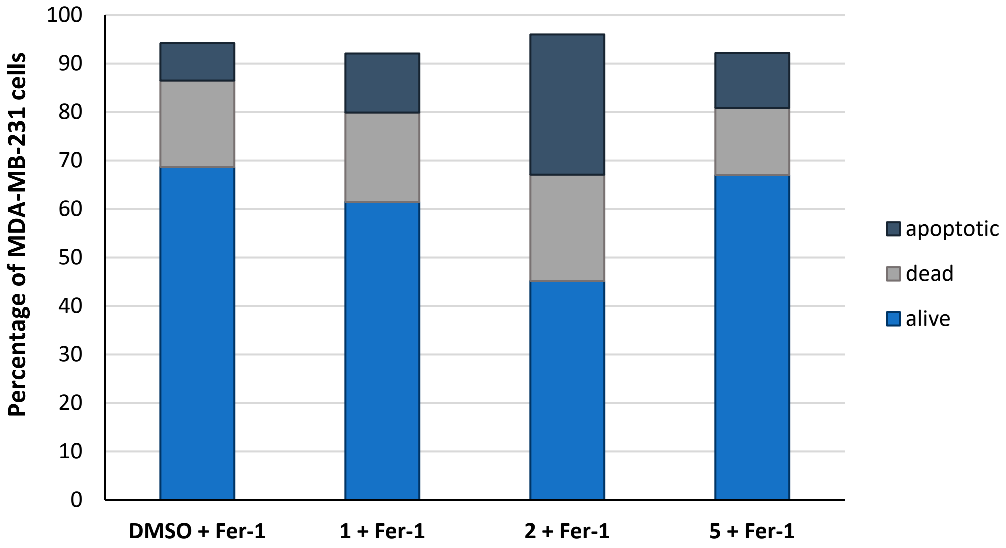

2.4 ROS

Figure S28: Induction of mROS in MDA-MB-231 cells after treatment with **1**, **2** and **5** (1 µM) for 24 h and staining with reducedMitoTrackerRed (red), wheat germ lectin (green) and Hoechst 33342 (blue).

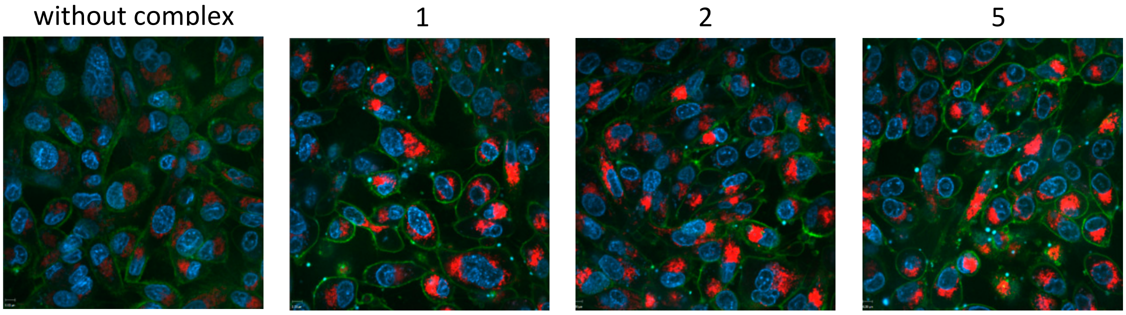

2.5 Mitochondrial membrane potential

Table S3: Mitochondrial membrane potential of A2780cis cells after a 24-hours incubation with **1**, **2** and **5** in concentrations of 0.1 µM and 0.5 µM (%); n = 2.

Complex	Mean ΔΨ at 0.1 µM (%)	Mean ΔΨ at 0.5 µM (%)
**1**	105.1 ± 11.7	52.9 ± 3.3
**2**	110.0 ± 9.4	56.7 ± 1.8
**5**	91.7 ± 4.3	69.7 ± 2.7

Table S4: Mitochondrial membrane potential of MDA-MB231 cells after a 24-hours incubation with **1**, **2** and **5** in concentrations of 0.1 µM and 0.5 µM (%); n = 2.

Complex	Mean ΔΨ at 0.1 µM (%)	Mean ΔΨ at 0.5 µM (%)
**1**	91.0 ± 7.8	53.9 ± 2.1
**2**	100.7 ± 1.2	65.1 ± 6.3
**5**	96.1 ± 20.3	54.4 ± 1.6

3 Cyclic voltammetry

3.1 Oxygen source for the formation of compound 7

Cyclic voltammetry measurements with traces of oxygen indicate that dioxygen can be a source for the formation of compound 7, however we wanted to investigate if water can also act as an oxygen donor. Therefore, we used complex 1 in a DMSO/water mixture under an argon atmosphere, which did not show any additional peaks, however with addition of an organic base (2-fluoropyridine) an additional reduction peak appeared.

Figure S29: Voltammogram of compound 1 (1 mM) in DMSO vs Fc (2 mM) with 0.03 ml water added to 3 ml DMSO solution.

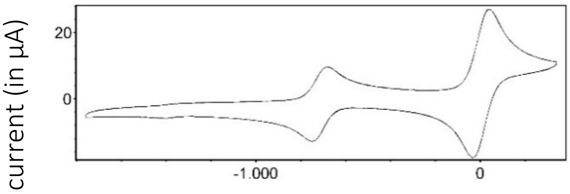

E vs. Fc (in DMSO)

Figure S30: Voltammogram of compound 1 (1 mM) with 2-fluoropyridine in DMSO vs Fc (2 mM) with 0.5 ml water added to 3 ml DMSO solution.

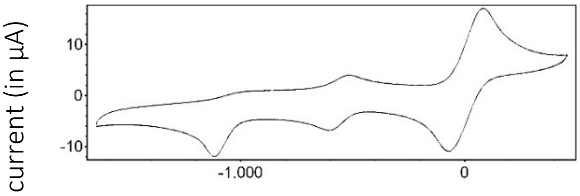

E vs. Fc (in DMSO)

Small concentrations of water (see **Figure 29**) slightly shifted reduction/oxidation potential and only a very small additional peak was found at -1272 mV (vs. Fc of water-free sample), however only at higher concentrations of water (see **Figure 30**) there was a big additional peak at -1182 mV (vs. Fc of water-free sample). There was no corresponding oxidation peak found. In contrast, compound 1 showed no additional signal, which means that 2-fluoropyridine plays a key role probably due to its basic properties in the formation of an additional reduction peak.

3.2 Voltammograms of the complexes **1–7**

3.2.1 Voltammograms of **1**

Figure S31: Voltammogram of **1** in DMSO.

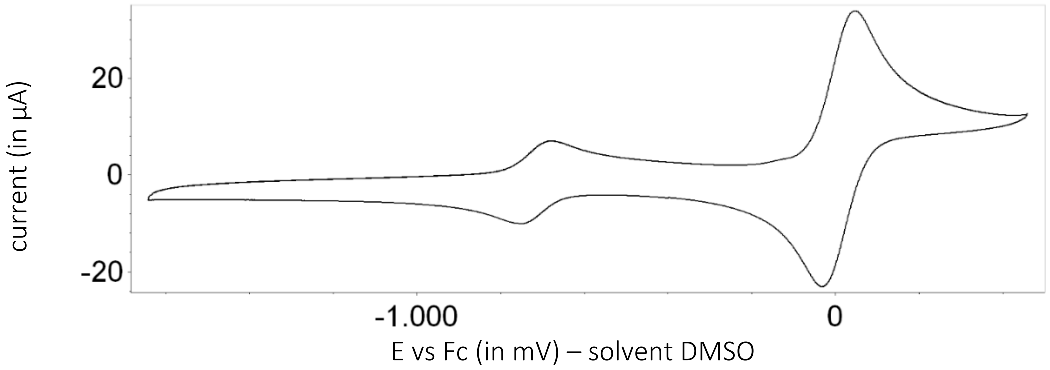

Figure S32: Voltammogram of **1** in DCM.

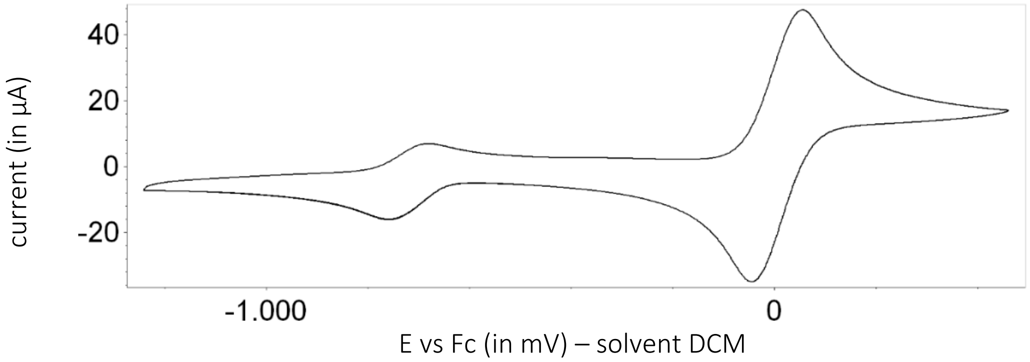

Figure S33: Voltammogram of **1** in ACN.

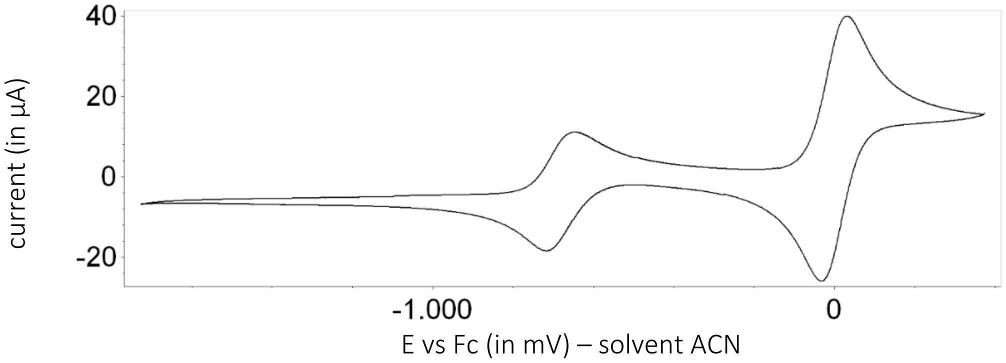

Figure S34: Voltammogram of **1** in DMF.

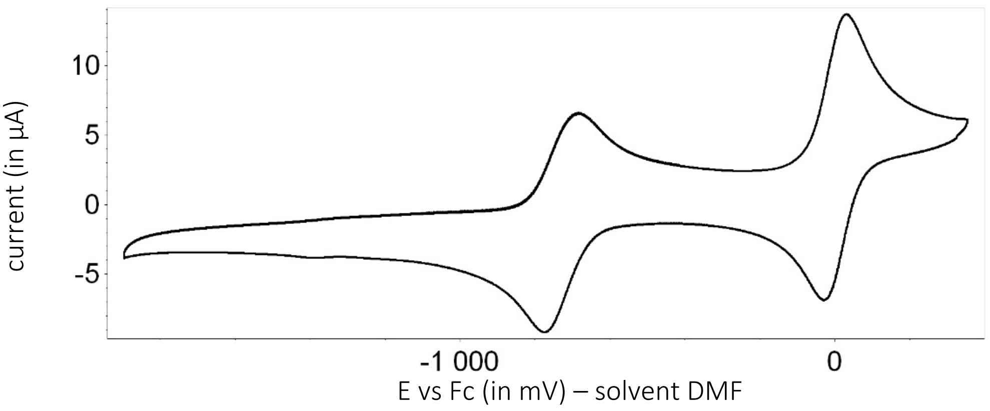

3.2.2 Voltammograms of **2**

Figure S35: Voltammogram of **2** in DMSO.

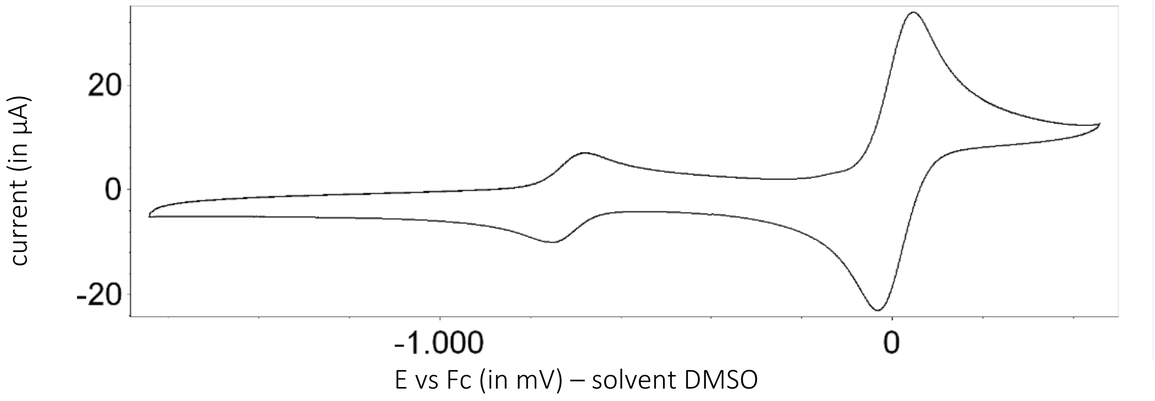

Figure S36: Voltammogram of **2** in DCM.

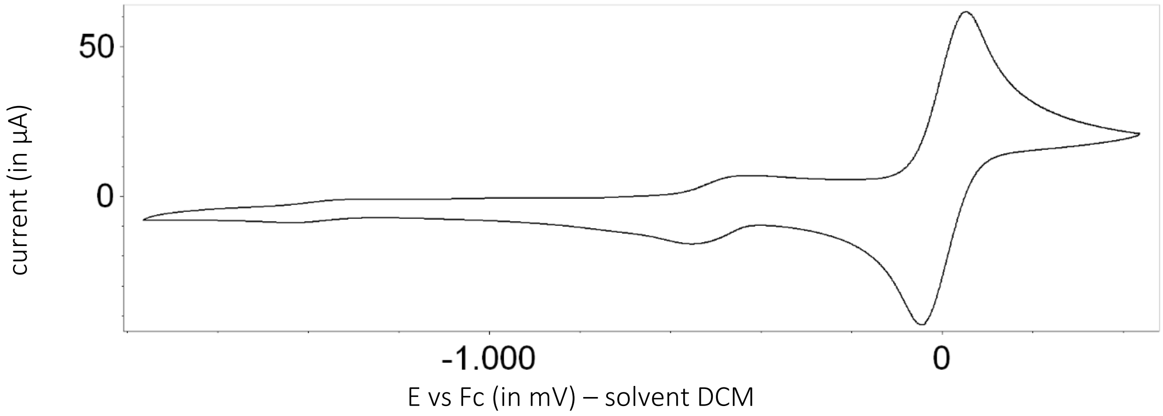

Figure S37: Voltammogram of **2** in ACN.

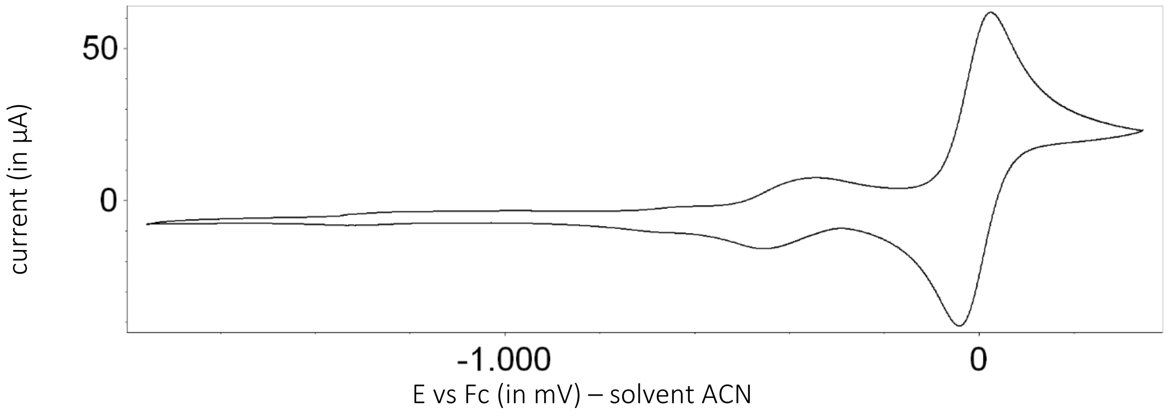

3.2.3 Voltammograms of **3**

Figure S38: Voltammogram of **3** in DMSO.

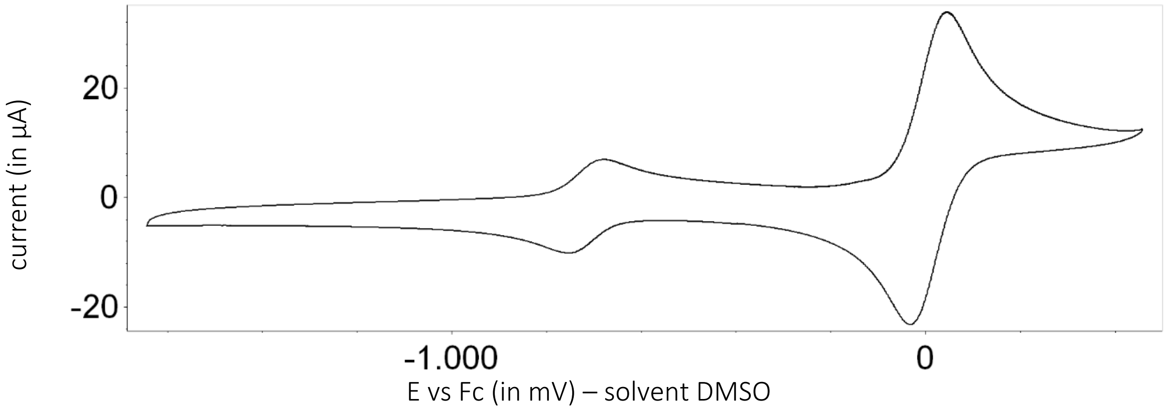

Figure S39: Voltammogram of **3** in DCM.

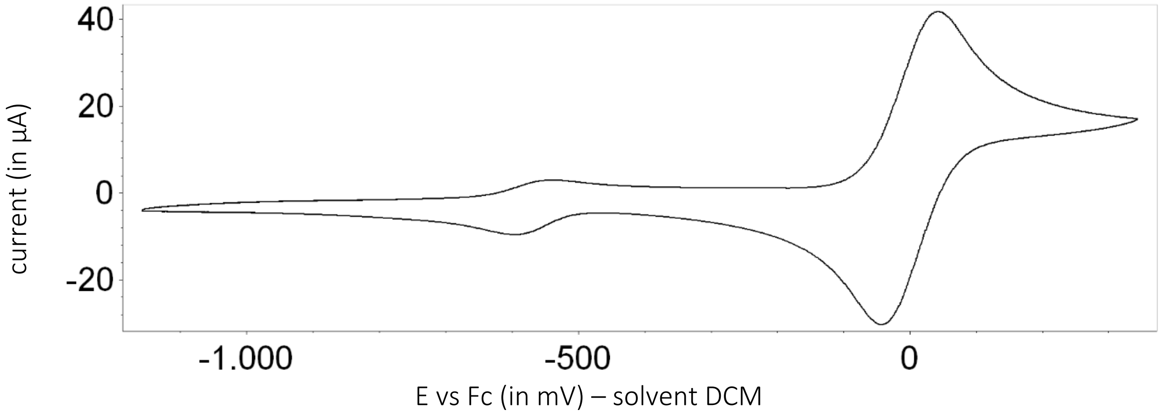

Figure S40: Voltammogram of **3** in ACN.

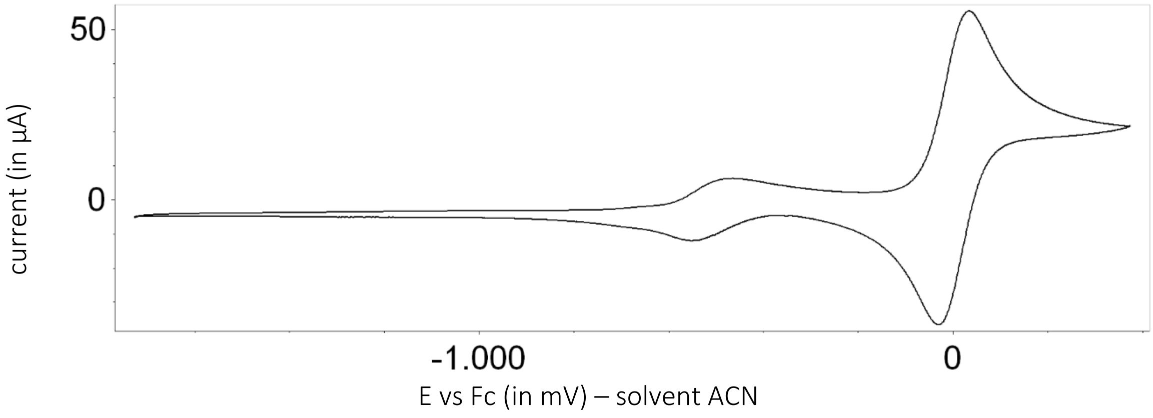

3.2.4 Voltammograms of **4**

Figure S41: Voltammogram of **4** in DMSO.

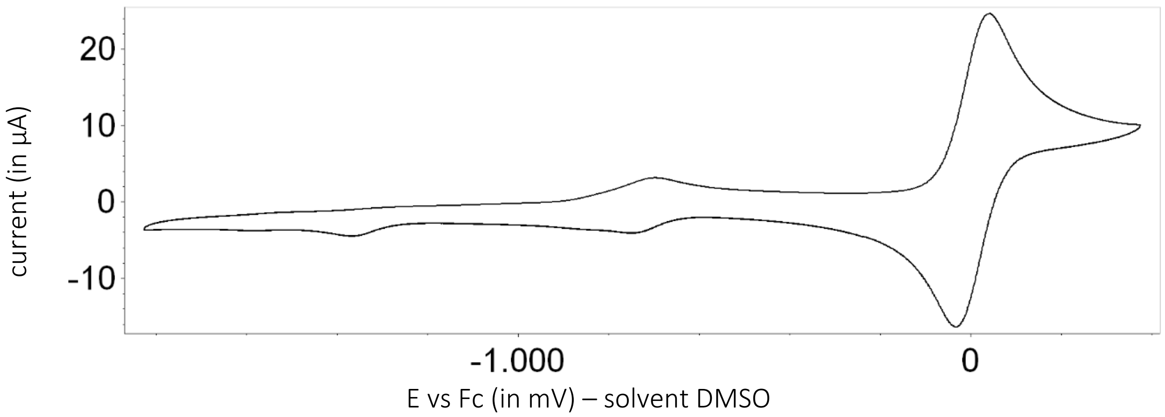

Figure S42: Voltammogram of **4** in DCM.

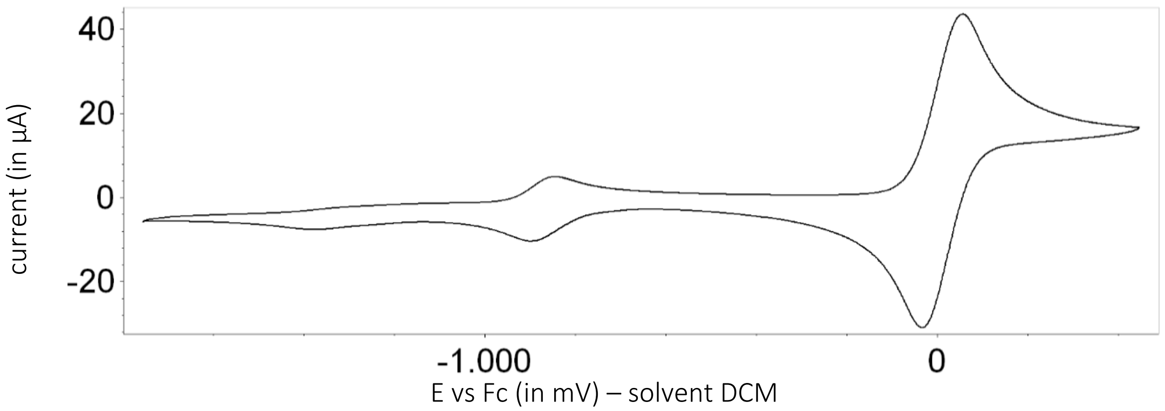

Figure S43: Voltammogram of **4** in ACN.

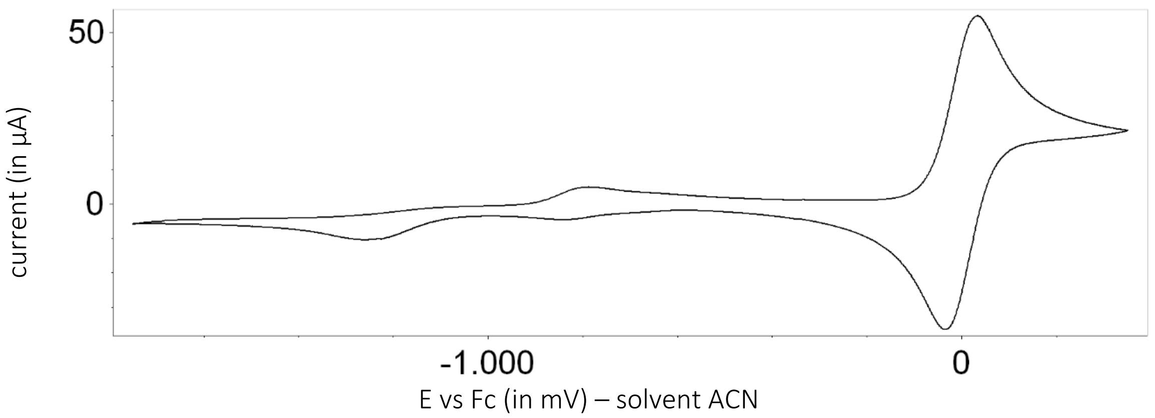

Figure S44: Voltammogram of **4** in DMF.

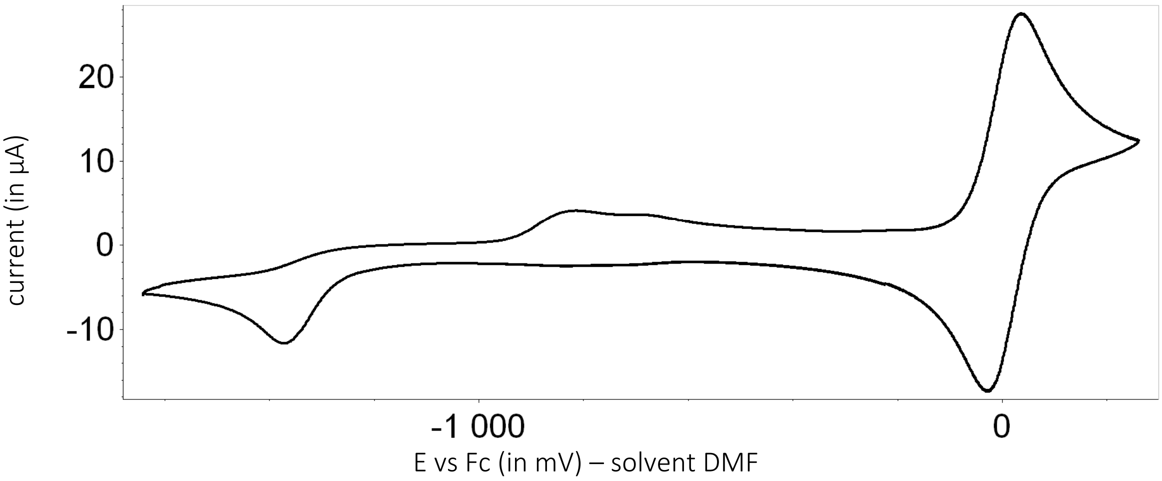

3.2.5 Voltammograms of **5**

Figure S45: Voltammograms of **5** in DMSO.

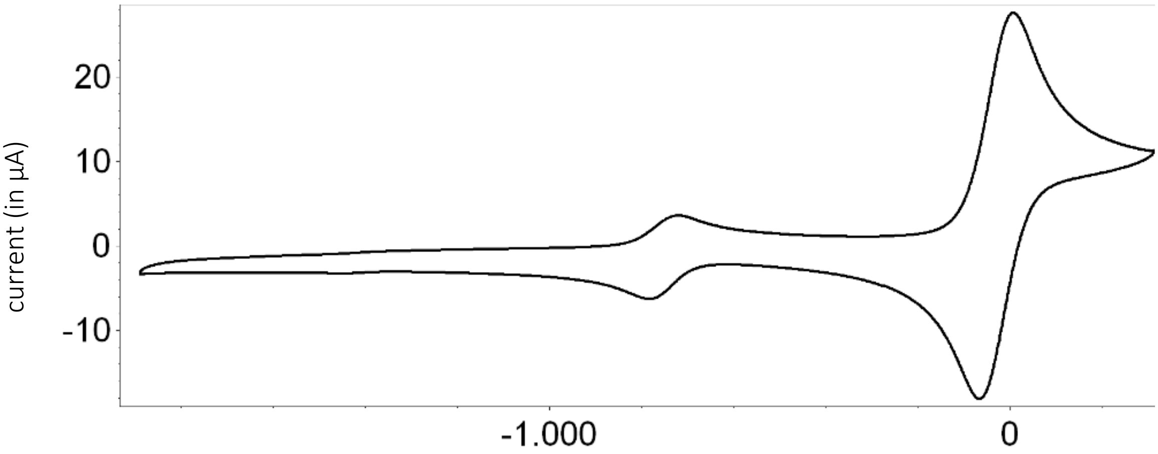

Figure S46: Voltammograms of **5** in DCM.

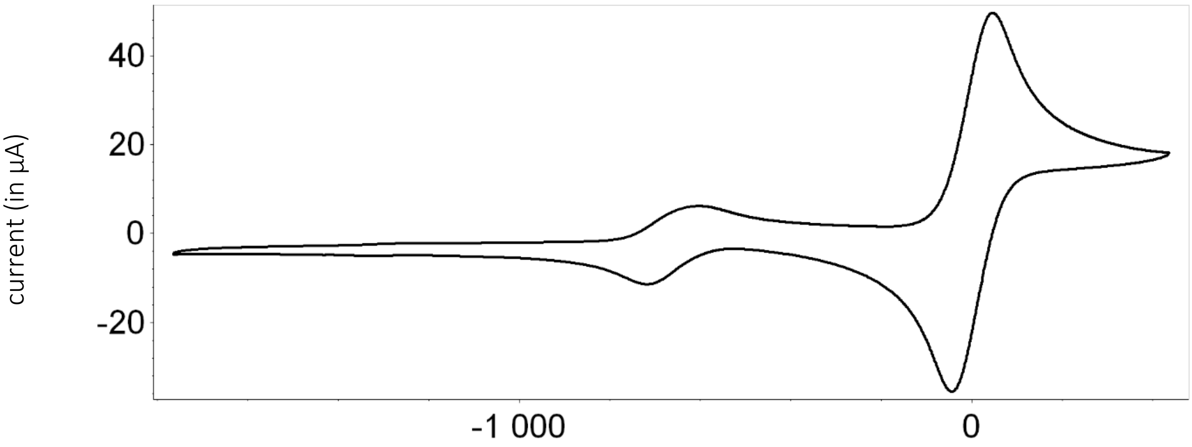

Figure S47: Voltammogram of **5** in ACN.

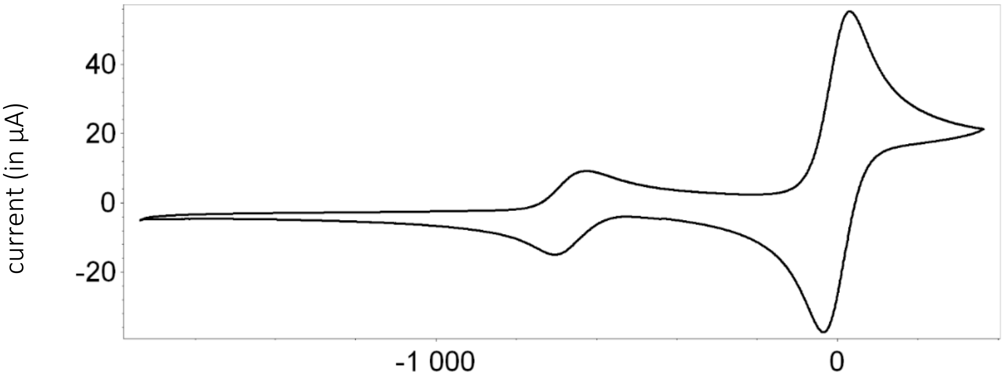

3.2.6 Voltammograms of **6**

Figure S48: Voltammogram of **6** in DMSO.

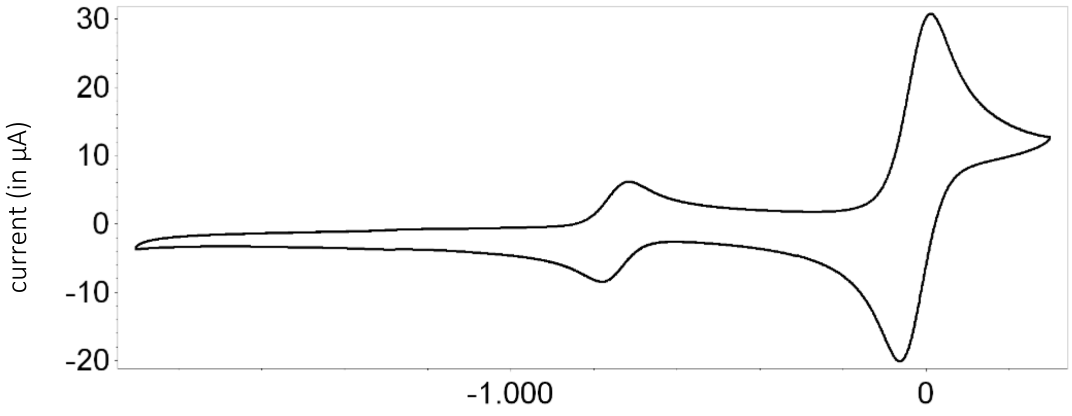

E vs Fc (in mV) – solvent DMSO

Figure S49: Voltammogram of **6** in DCM.

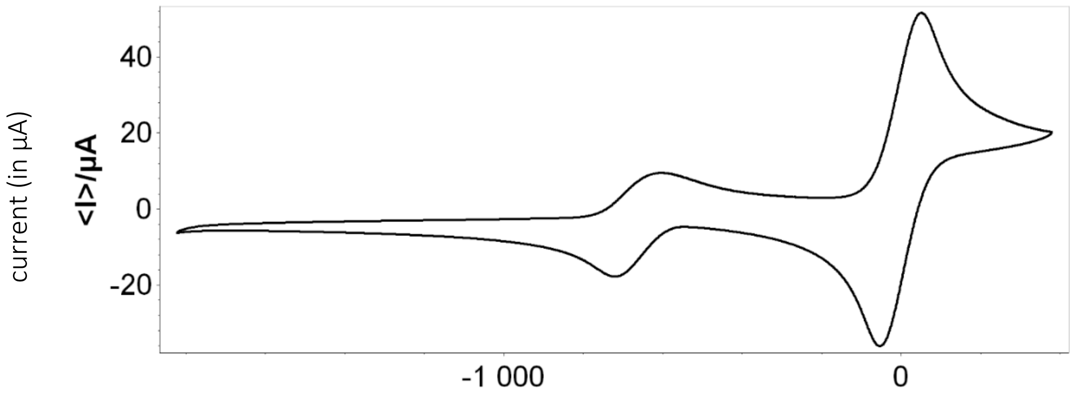

E vs Fc (in mV) – solvent DCM

Figure S50: Voltammogram of **6** in ACN.

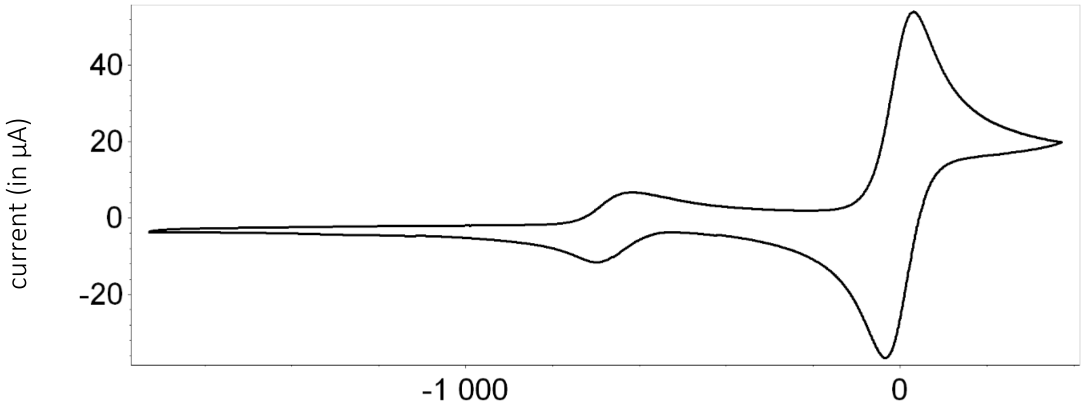

E vs Fc (in mV) – solvent ACN

3.2.7 Voltammograms of **7**

Figure S51 Voltammogram of **7** in DMSO.

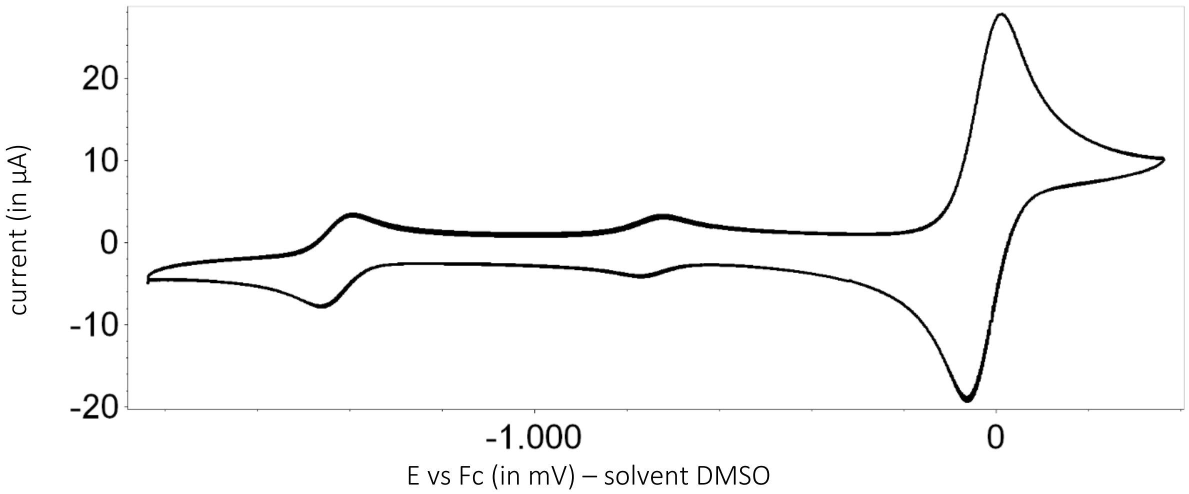

Figure S52: Voltammogram of **7** in DCM.

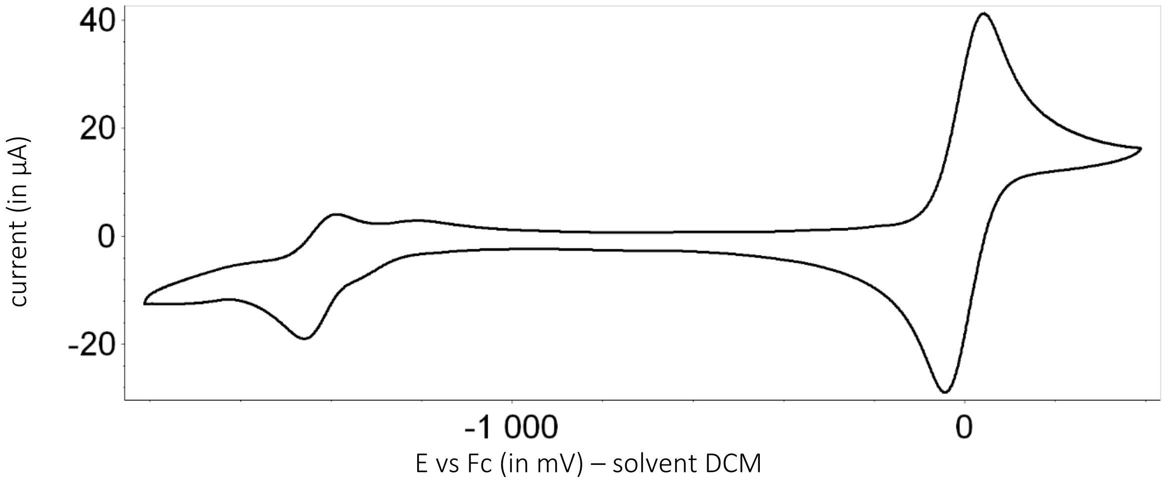

Figure S53: Voltammogram of **7** in ACN.

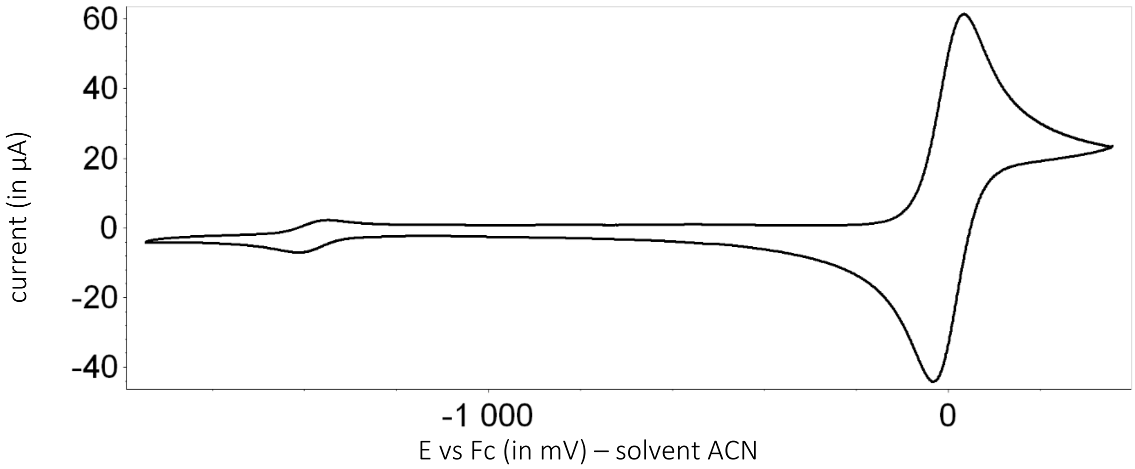

Figure S54: Voltammogram of **7** in DMF.

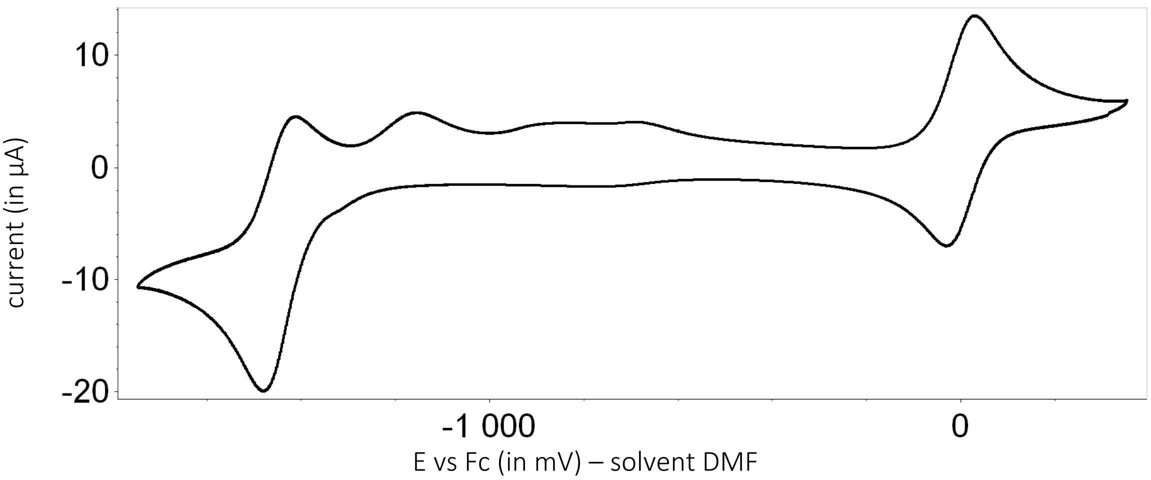

Supplement: Supplementary file 1 [file ijms-24-02173-s001.zip › ijms-2074218-supplementary.pdf]
